# Maintenance of aversive memories shown by fear extinction-impaired phenotypes is associated with increased activity in the amygdaloid-prefrontal circuit

**DOI:** 10.1038/srep21205

**Published:** 2016-02-15

**Authors:** Daniela Laricchiuta, Luana Saba, Paola De Bartolo, Silvia Caioli, Cristina Zona, Laura Petrosini

**Affiliations:** 1 IRCCS Fondazione Santa Lucia, Rome, Italy; 2 Department of Psychology, University Sapienza of Rome, Rome, Italy; 3 Department of Neuroscience, University of Rome “Tor Vergata”, Rome, Italy; 4 Department of Sociological and Psychopedagogical Studies, University Guglielmo Marconi of Rome, Rome, Italy

## Abstract

Although aversive memory has been mainly addressed by analysing the changes occurring in average populations, the study of neuronal mechanisms of outliers allows understanding the involvement of individual differences in fear conditioning and extinction. We recently developed an innovative experimental model of individual differences in approach and avoidance behaviors, classifying the mice as Approaching, Balancing or Avoiding animals according to their responses to conflicting stimuli. The approach and avoidance behaviors appear to be the primary reactions to rewarding and threatening stimuli and may represent predictors of vulnerability (or resilience) to fear. We submitted the three mice phenotypes to Contextual Fear Conditioning. In comparison to Balancing animals, Approaching and Avoiding mice exhibited no middle- or long-term fear extinction. The two non-extinguishing phenotypes exhibited potentiated glutamatergic neurotransmission (spontaneous excitatory postsynaptic currents/spinogenesis) of pyramidal neurons of medial prefrontal cortex and basolateral amygdala. Basing on the *a priori* individuation of outliers, we demonstrated that the maintenance of aversive memories is linked to increased spinogenesis and excitatory signaling in the amygdala-prefrontal cortex fear matrix.

Advances in the understanding of fear memories have been made through studies on fear conditioning that entails pairing a Conditioned Stimulus (CS, e.g., tone or context) with an Unconditioned Stimulus (US, e.g., foot-shock) to elicit a fear Conditioned Response (CR, e.g., freezing)^1^. The phase during which the association between the CS and US is established, called “conditioning,” is followed by a phase of “consolidation” during which the fear mnemonic trace is stabilized^2^,^3^,^4^. When the consolidated memory trace is recalled (“retrieval”), it returns to a labile state, thereby making it sensitive to change and disruption^5^. Short re-exposure to the CS in the absence of the US elicits the CR and, at the same time, initiates a new process of memory trace elaboration (“reconsolidation”). Repeated re-exposure to the CS alone gradually weakens the engram (“extinction”)^6^. Thus, the reconsolidation integrates new information into the original mnemonic trace, thereby strengthening or weakening the fear memory^7^. In the latter case, the extinction does not directly modify the original fear memory but results in the formation of new associations (CS – no US) that compete with the original engram to mask it. Thus, extinction implies new learning.

In humans, improper extinction is particularly relevant for trauma-related psychopathologies, such as post-traumatic stress disorder (PTSD), major depressive disorder, generalized anxiety disorder and phobias^8^. A central point of these deficits is that only a minority of exposed-to-trauma individuals develop significant and prolonged fear symptomatology^9^. Given that it is still very difficult to study neuronal signaling related to improper fear inhibition in humans, only an experimental outlier-based approach can produce insights into the neurobiological correlates of impaired fear extinction. The present study evaluates how phenotypes characterized by individual differences in approach and avoidance behaviors^10^,^11^,^12^ (ranging from normal to outliers) react to fear conditioning, reconsolidation, and extinction. Approach and avoidance are the primary reactions to rewarding and threatening stimuli, on which all successive responses are based to achieve successful adaptation^13^. Individual differences in approach and avoidance may represent predictors of vulnerability (or resilience) to fear, stress and anxiety. Excessive approach or avoidance behaviors can result in psychopathological disorders, including anxiety and PTSD, on one hand, or depression and substance abuse, on the other hand^14^.

The present research associates behavioral responses to fear with electrophysiological and morphological correlates in the “fear matrix” of the brain, whereby control centers, integrative sites and effector sites can be distinguished^15^. Specifically, during the fear conditioning, reconsolidation, and extinction phases, the prelimbic (PL) and infralimbic (IL) subregions of the control center represented by the medial prefrontal cortex (mPFC) receive from and project back to the integrative site in the basolateral amygdala (BLA)^16^. This region in turn projects to the central nucleus (CE) of the amygdala directly or indirectly via inhibitory intercalated cells (ITC). Finally, from the output neurons of the CE, information is relayed to the effector sites in the periaqueductal gray (PAG) and pre-motor structures that mediate fear CR^17^,^18^.

Within the amygdala, cannabinoid-type 1 (CB_1_) receptors are abundant and presynaptically present only on GABAergic interneurons (and not on GABAergic ITC and glutamatergic pyramidal cells)^19^. It has been shown that individuals with trauma-related psychopathology, such as PTSD, have higher brain-wide CB_1_ availability in comparison to healthy controls with or without histories of trauma^20^. Furthermore, in individuals with trauma-related psychopathologies, greater CB_1_ availability in the amygdala has been associated with increased attentional bias to threat and increased severity of threat symptomatology^21^. These observations crucially indicate that enhanced CB_1_ signaling is implicated in trauma-related deficits.

Until now, in the experimental studies, heterogeneous populations of animals have been *a posteriori* disaggregated based upon their susceptibility to fear^22^ and their ability to extinguish fear^23^,^24^,^25^, even through instrumental responses^26^. Surprisingly, we realized that our model of individual differences in approach and avoidance behaviors could provide the first experimental evidence for the *a priori* CB_1_ pattern being linked to trauma-related deficits. In fact, we demonstrated that the individual differences among mice classified as approaching (AP), balancing (BA) or avoiding (AV) animals are mirrored by different tuning of CB_1_ signaling at the brain-wide level^11^,^12^. Interestingly, in AP and AV mice, CB_1_ density and functionality are increased just in the amygdala^11^. Thus, we hypothesize that when submitted to a Contextual Fear Conditioning (CFC) test, AP and AV mice may exhibit improper fear extinction associated with altered electrophysiological and morphological correlates in the “fear matrix”.

## Results

### Analyses of middle-term extinction processes

#### CFC

**Figure 1:**
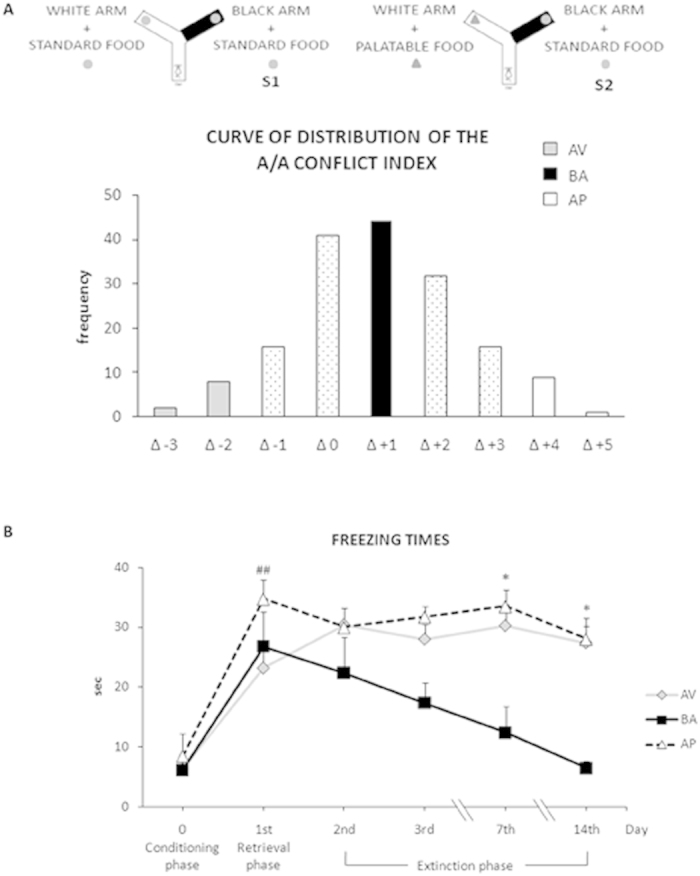
The Approach/Avoidance (A/A) conflict index permits to select the avoiding (AV) and approaching (AP) animals that do not extinguish middle-term fear memories, in comparison to balancing (BA) animals. (**A**) In the upper part of the Figure the A/A Y-Maze and its two sessions (S1: first session; S2: second session) are illustrated. A/A conflict index represents the difference (Δ) in the number of white choices between sessions. (**B**) Freezing times of AV, BA and AP phenotypes were similar in Conditioning and Retrieval phases, and they significantly increased between Conditioning and Retrieval phases (## at least P = 0.001). On day 14^th^ the freezing times of AV and AP mice still did not significantly differ from those of Retrieval phase and were significantly higher than those of BA animals at day 7^th^ and 14^th^ (* at least P = 0.03). From day 7^th^ on, the freezing times of BA mice were similar to those of Conditioning phase. Data are presented as means ± SEM.

Fifteen animals (5 mice/phenotype) (Fig. 1A) were submitted to CFC with repetitive sessions at day 0 (Conditioning), 1^st^ (Retrieval), 2^nd^, 3^rd^, 7^th^, and 14^th^ (Extinction). AV, BA and AP animals showed similar responses in the Conditioning phase (AV *vs.* BA: P = 0.97; AV *vs.* AP: P = 0.76; BA *vs.* AP: P = 0.75) and similar consolidation processes of aversive memories in the Retrieval phase (AV *vs.* BA: P = 0.59; AV *vs.* AP: P = 0.14; BA *vs.* AP: P = 0.30). BA mice progressively extinguished fear memories over time and, from day 7^th^ their freezing times returned to a similar level to during the Conditioning phase. Interestingly, no extinction of fear memories was observed in AV and AP phenotypes. At day 14^th^ the freezing times of AV (P = 0.38) and AP (P = 0.20) mice did not significantly differ from those of the Retrieval phase. Both AV and AP phenotypes showed freezing times that were similar during the entire task and higher than those of BA animals at days 7^th^ and 14^th^. A two-way ANOVA (phenotype x day) of freezing times revealed significant phenotype (F_2,12_ = 10.89; P = 0.0021) and day (F_5,60_ = 18.67; P < 0.0001) effects. The interaction was also significant (F_10,60_ = 2.98; P = 0.004). Significant post-hoc comparisons on interaction are shown in Fig. 1B.

### Electrophysiological results

**Figure 2:**
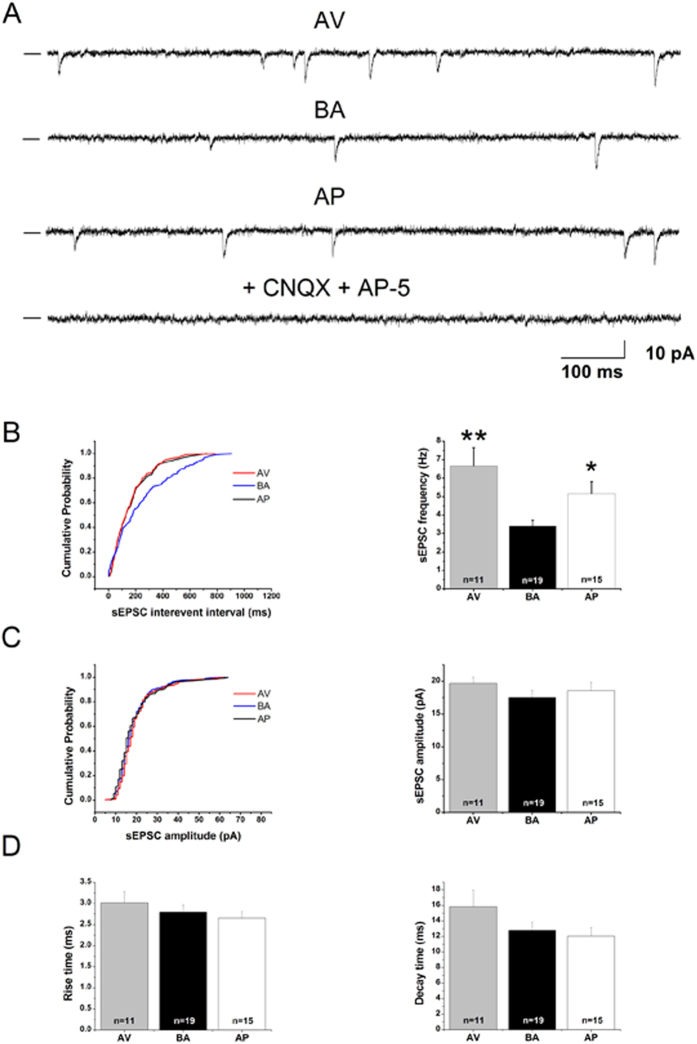
Prefrontal pyramidal neurons of avoiding (AV) and approaching (AP) animals show a significant increase in the spontaneous excitatory postsynaptic currents (sEPSCs) frequencies compared to neurons of balancing (BA) animals. (**A**) Representative traces of sEPSCs recorded from prefrontal pyramidal neurons of AV, AP and BA animals. In the presence of 6-cyano-7-nitroquinoxyline-2,3-dione (CNQX, 10 μM) and *amino*-5-phosphonovaleric *acid* (AP-5, 50 μM) ionotropic glutamatergic receptor antagonists, the excitatory synaptic activity was completely abolished. (**B**) On the left, cumulative probability plots of sEPSC inter-event intervals in pyramidal neurons of AP, AV and BA mice. In the AV and AP mice the inter-event intervals were significantly lower compared to those of BA mice (P < 0.05; K–S test). On the right, the sEPSC frequencies of pyramidal neurons of AV, AP and BA mice are reported. (**C**) On the left, cumulative probability plots of sEPSC amplitude in pyramidal neurons of AP, AV and BA mice (P > 0.05; K–S test). On the right, the sEPSC amplitudes of pyramidal neurons of AV, AP and BA mice are reported. (**D**) sEPSC rise time (left) and decay time (right) of pyramidal neurons of AV, AP and BA mice. **P < 0.01; *P < 0.05. Data are presented as means ± SEM. Numbers inside the bar plots indicate the number of pyramidal neurons recorded (at least 2–3 for each AV, BA and AP animal).

Spontaneous excitatory postsynaptic currents (sEPSCs) in mPFC pyramidal neurons were recorded in the three phenotypes and were completely abolished when an AMPA/Kainate receptor antagonist (6-cyano-7-nitroquinoxyline-2,3-dione, CNQX) and a NMDA receptor antagonist (*amino*-5-phosphonovaleric *acid*, AP-5) were added to the artificial cerebrospinal fluid (ACSF) solution, thereby confirming that synaptic events were mediated by ionotropic glutamatergic receptors (Fig. 2A). Analysis of the sEPSCs demonstrated that the mean inter-event interval was significantly shorter in neurons in AV and AP mice than in BA mice, thereby indicating that neurons in AV and AP animals exhibited an increased sEPSC frequency in comparison to neurons in BA animals, as indicated by post-hoc comparisons on one-way ANOVA of frequency values (F_2,12_ = 5.25; P = 0.02). The frequencies of sEPSC exhibited by AV and AP mice were not significantly different (P = 0.06) (Fig. 2B). Conversely, a one-way ANOVA of sEPSC amplitude failed to reveal a significant phenotype effect (F_2,12_ = 0.16; P = 0.85) (Fig. 2C). Furthermore, analyses of current kinetics (Fig. 2D) showed that the rise time (F_2,12_ = 1.14; P = 0.35) and decay time (F_2,12_ = 1.31; P = 0.31) were not significantly different among neurons from AV, AP and BA mice.

### Morphological reconstruction of pyramidal mPFC neurons

**Figure 3:**
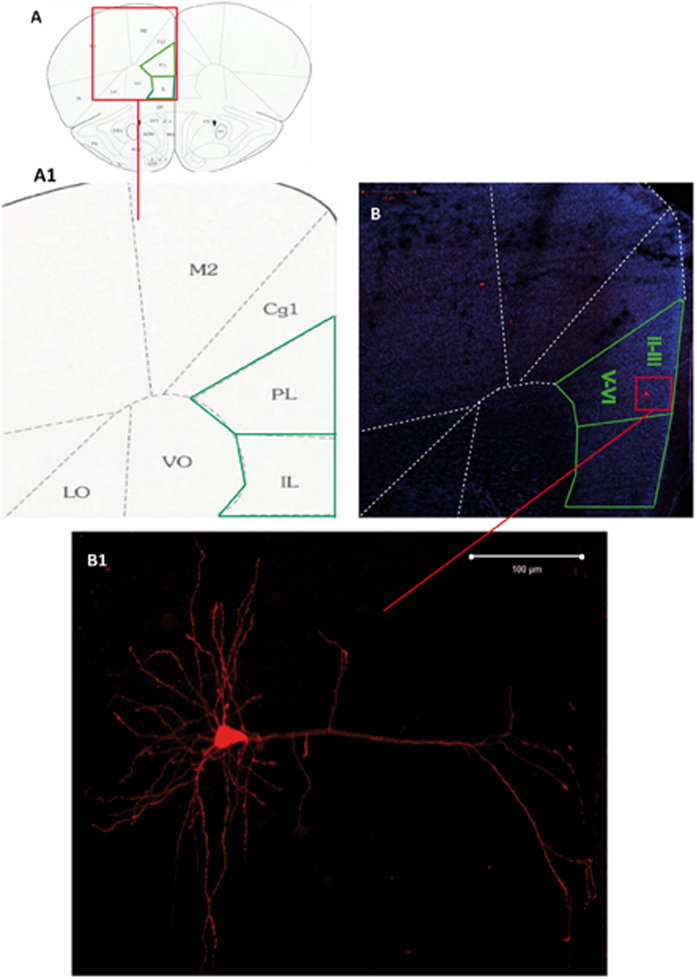
Morphological reconstruction of a recorded prefrontal pyramidal neuron by biocytin labelling. (**A)-(A1**) Schematic drawings showing the localization of PreLimbic (PL) - InfraLimbic (IL) medial prefrontal cortex delimited by green lines. (**B)** Low-magnification picture showing a recorded biocytin-filled neuron (red box) of the II-III layer in the PL medial prefrontal cortex. (B1) Confocal microscope image of the same biocytin-filled pyramidal neuron depicted in the panel (**B**). M2: secondary Motor Cortex; Cg1: Cingulate Cortex 1; VO: Ventral Orbital Cortex; LO: Lateral Orbital Cortex. Scale bars: Panel (**B**): 50 μm; Panel B1: 100 μm. II-III, V-VI = PL cortical layers.

All recorded neurons filled with biocytin in fluorescent Nissl-stained slices were located in layer II-III of the mPFC area and exhibited the morphological features of cortical pyramidal neurons, as described in the Methods (Fig. 3).

### Results of analyses of long-term extinction processes

#### CFC

**Figure 4:**
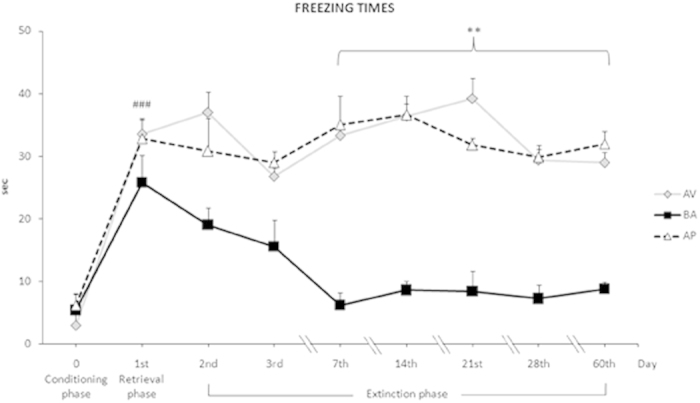
Avoiding (AV) and approaching (AP) animals do not extinguish long-term fear memories, in comparison to balancing (BA) animals. Freezing times of AV, BA and AP phenotypes were similar in Conditioning and Retrieval phases, and they significantly increased between Conditioning and Retrieval phases (### at least P = 0.0006). On day 60^th^ the freezing times of AV and AP mice still did not significantly differ from those of Retrieval phase and were significantly higher than those of BA animals from day 7^th^ to 60^th^ (** at least P = 0.01). From day 7^th^ on, the freezing times of BA mice were similar to those of Conditioning phase. Data are presented as means ± SEM.

Fifteen animals (5 mice/phenotype) were submitted to a CFC protocol with a long-term extinction phase (days 2^nd^, 3^rd^, 7^th^, 14^th^, 21^st^, 28^th^ and 60^th^). Once again, AV, BA and AP animals showed similar responses in the Conditioning phase (AV *vs.* BA: P = 0.83; AV *vs.* AP: P = 0.79; BA *vs.* AP: P = 0.94) and similar consolidation processes of aversive memories in the Retrieval phase (AV *vs.* BA: P = 0.52; AV *vs.* AP: P = 0.94; BA *vs.* AP: P = 0.57). BA mice progressively extinguished fear memories over time. In fact, from day 7^th^ to 60^th^ BA animals exhibited freezing times that were similar to those they had displayed during the Conditioning phase. Conversely, even at day 60^th^, AV and AP animals had not extinguished fear memories. Namely, at day 60^th^ the freezing times of AV (P = 0.46) and AP (P = 0.87) mice did not significantly differ from those of the Retrieval phase. During the entire task, both AV and AP phenotypes showed freezing times that were similar to each other and significantly higher than those of BA animals from days 7^th^ to 60^th^. A two-way ANOVA (phenotype x day) of freezing times revealed significant phenotype (F_2,12_ = 17.36; P = 0.0003) and day (F_8,96_ = 12.13; P < 0.0001) effects. The interaction was also significant (F_16,96_ = 2.85; P = 0.0008). Significant post-hoc comparisons on interaction are shown in Fig. 4.

### Morphological results

**Figure 5:**
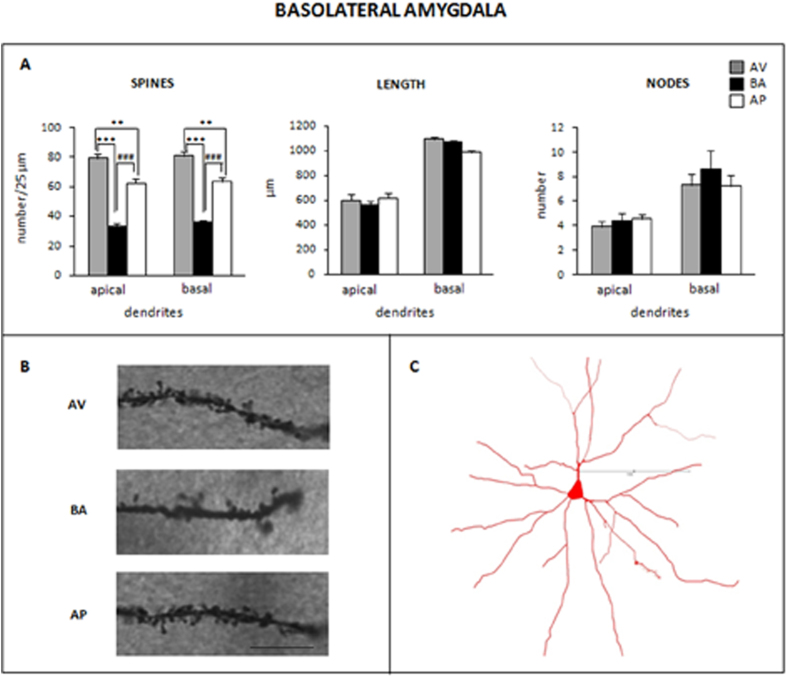
Morphological pattern of basolateral amygdala pyramidal neurons. (**A)** Mean values of spine density (number/25 μm), length (μm) and nodes of apical and basal dendrites of avoiding (AV), balancing (BA) and approaching (AP) mice (**P ≤ 0.002; ***^, ###^P = 0.0002). Data are presented as means ± SEM. (**B)** Photomicrographs visualizing the spines of representative apical dendritic segments of pyramidal neurons of basolateral amygdala in AV, BA and AP mice. Magnification: 1000X. Scale bar: 10 μm. (**C)** Camera lucida drawing of a representative pyramidal neuron of basolateral amygdala. Scale bar: 100 μm.

**Figure 6:**
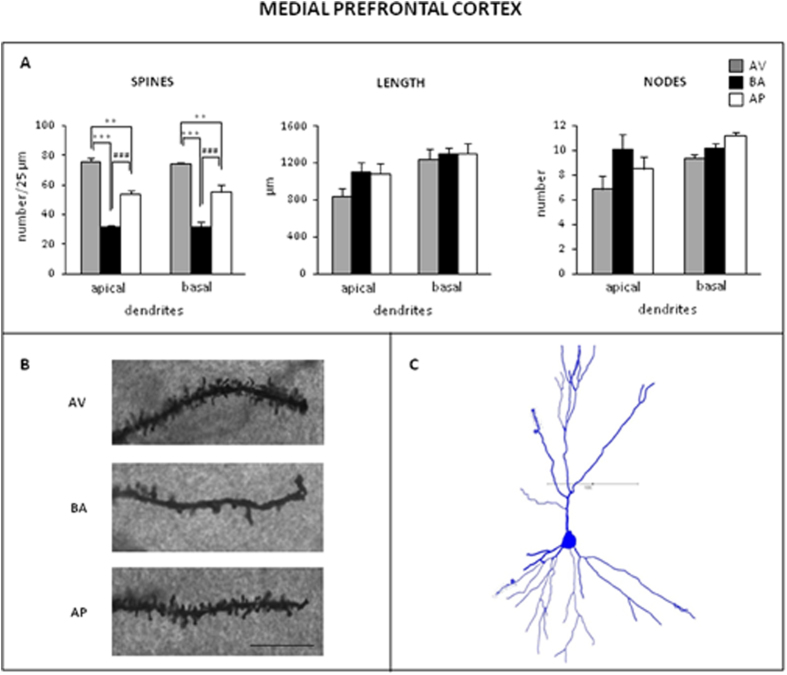
Morphological pattern of medial prefrontal cortex pyramidal neurons. (**A)** Mean values of spine density (number/25 μm), length (μm) and nodes of apical and basal dendrites of avoiding (AV), balancing (BA) and approaching (AP) mice (***^, ###^P ≤ 0.0005). Data are presented as means ± SEM. (**B)** Photomicrographs visualizing the spines of representative apical dendritic segments of pyramidal neurons of medial prefrontal cortex in AV, BA and AP mice. Magnification: 1000X. Scale bar: 10 μm. (**C)** Camera lucida drawing of a representative pyramidal neuron of medial prefrontal cortex. Scale bar: 100 μm.

AV, BA and AP phenotypes exhibited different spine densities and similar dendritic branching of apical and basal arborizations of BLA (Fig. 5) and IL/PL mPFC (Fig. 6) pyramidal neurons.

#### BLA

As for apical dendrites, post-hoc comparisons of the significant ANOVA of spine density (F_2,12_ = 66.35; P < 0.0001) indicated that AV animals had the highest spine density in comparison to AP (P = 0.001) and BA animals (P = 0.0002). Even AP mice had a spine density that was higher (P = 0.0002) than that of BA animals. ANOVAs of dendritic length (F_2,12_ = 0.41; P = 0.67) and nodes (F_2,12_ = 0.52; P = 0.61) did not reveal a significant difference among phenotypes.

As for basal dendrites, post-hoc comparisons of significant ANOVA of spine density (F_2,12_ = 49.85; P < 0.0001) indicated that AV animals showed the highest spine density in comparison to AP (P = 0.002) and BA animals (P = 0.0002). Even AP animals had a spine density that was higher (P = 0.0002) than that of BA animals. ANOVAs of dendritic length (F_2,12_ = 0.26; P = 0.77) and nodes (F_2,27_ = 0.43; P = 0.66) did not reveal a significant effect.

#### mPFC

As for apical dendrites, post-hoc comparisons of significant ANOVA of spine density (F_2,12_ = 73.97; P < 0.0001) indicated that AV animals had the highest spine density in comparison to AP and BA animals (P = 0.0002). Even AP animals had a spine density that was higher (P = 0.0002) than that of BA animals. ANOVAs of dendritic length (F_2,12_ = 2.47; P = 0.13) and nodes (F_2,12_ = 2.35; P = 0.14) did not reveal a significant difference among phenotypes.

As for basal dendrites, post-hoc comparisons of significant ANOVA of spine density (F_2,121_ = 59.63; P < 0.0001) indicated that AV animals had the highest spine density in comparison to AP (P = 0.0005) and BA animals (P = 0.0002). Even AP animals had a spine density that was higher (P = 0.0002) than that of BA animals. ANOVAs of dendritic length (F_2,12_ = 0.18; P = 0.83) and nodes (F_2,12_ = 1.14; P = 0.35) did not reveal a significant effect.

## Discussion

The neurobiology of fear memory and extinction has been typically studied by analyzing changes that occur in average populations^18^. However, the study of neuronal mechanisms that characterize the outliers allows us to study trauma-related diseases^27^. Following this approach, we investigated fear extinction and its neuronal correlates in outlier animals that are characterized by approach and avoidance behaviors that we have previously demonstrated to be associated with different tuning of CB_1_ signaling in the amygdala^11^.

Although AV, BA and AP phenotypes exhibited similar behaviors in the Conditioning and Retrieval phases, only BA mice reduced their freezing times over time. Indeed, no fear extinction was observed in the AV and AP groups, even at day 60^th^ of CFC. In parallel, AV and AP mice exhibited increased excitatory neurotransmission in mPFC pyramidal neurons, in comparison to BA animals. The higher sEPSC frequency was not associated with different sEPSC amplitude or kinetic properties (rise and decay times). Such an electrophysiological pattern indicates increased glutamate release at the presynaptic level and no change in the postsynaptic features of the pyramidal neurons. Thus, the modified glutamatergic neurotransmission in the pyramidal neurons of AV and AP phenotypes might be related to increased excitatory afferents reaching the mPFC. This finding is nicely consistent with the high spine density of mPFC layer II-III pyramidal neurons of AV and AP mice. Similarly, enhanced spinogenesis was observed in BLA pyramidal neurons. Notably, it has been demonstrated that in mouse cortical layer II-III pyramidal neurons, glutamatergic signals trigger growth of new spines that express glutamatergic receptors and are rapidly functional^28^. Thus, the lack of fear extinction, as shown by the outlier AV and AP mice, was associated with aberrant neuronal activation in amygdaloid-prefrontal circuit that allows us to advance a possible neuronal substrate that is linked to the rigid maintenance of aversive memories.

**Figure 7:**
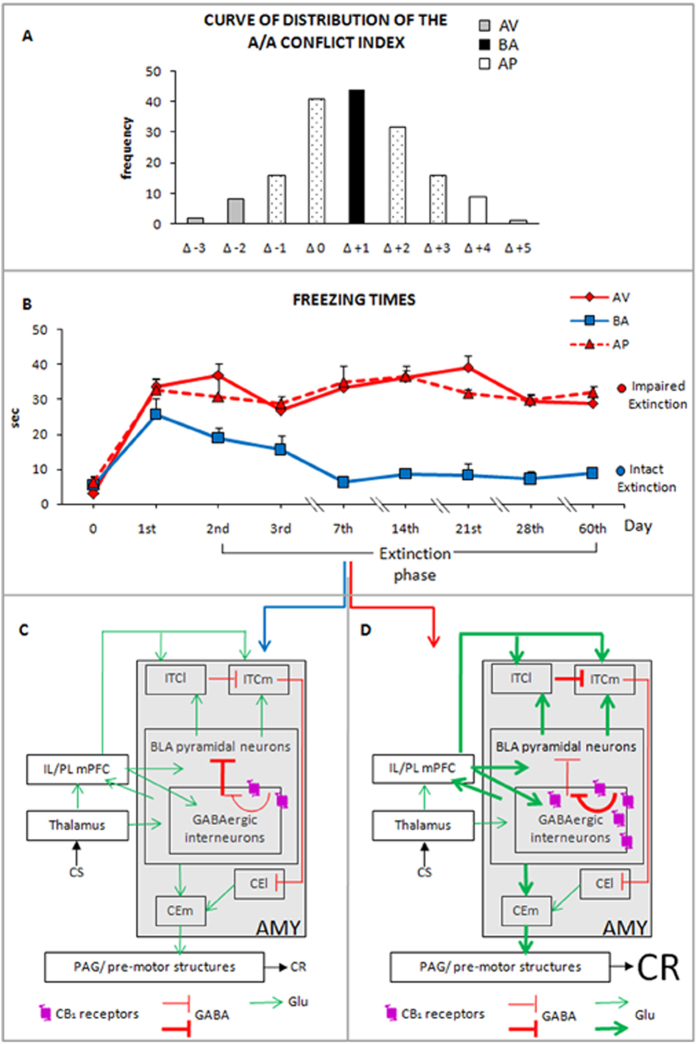
Behavioral and neuronal correlates of individual differences in fear extinction: extinguishing *vs.* non- extinguishing mice. (**A**) Curve of distribution of the Approach/Avoidance (A/A) conflict index that allowed categorizing the avoiding (AV), balancing (BA) and approaching (AP) mice. (**B**) In comparison to BA animals, AV and AP mice do not extinguish fear memories. (**C**) Intact fear extinction is associated with recruitment of reciprocal cortico-amygdala (Infralimbic and Prelimbic medial Prefrontal Cortex: IL/PL mPFC, small white box; Amygdala: AMY, large gray box) connections (arrows and segments). Namely, thalamic and cortical (IL/PL mPFC) glutamatergic projections convey the Conditioned Stimulus (CS) signals on the basolateral amygdala (BLA) pyramidal neurons and GABAergic interneurons. BLA pyramidal neurons relay the CS signals to medial Intercalated Cells (ITCm) that in turn inhibit the amygdaloid lateral central nucleus (CEl) that projects to the amygdaloid medial central nucleus (CEm). BLA pyramidal neurons excite also the lateral Intercalated Cells (ITCl), which inhibit the ITCm. Ultimately, such network elicits the disinhibition of the CEl neurons and the activation of the CEm. The pyramidal neurons of the IL/PL mPFC project not only to the BLA but also to the ITCl and ITCm. Within the BLA, GABAergic interneurons that inhibit the glutamatergic pyramidal neurons, contain the cannabinoid-type 1 (CB_1_) receptors that presynaptically inhibit GABA release. Finally, from the amygdaloid CEm, information is relayed to the Periaqueductal Gray (PAG) and pre-motor structures that mediate fear Conditioned Responses (CR). (**D**) AV and AP animals have increased availability of the amygdaloid CB_1_ receptors that might disinhibit the BLA pyramidal neurons and increase the output to mPFC pyramidal neurons. mPFC pyramidal neurons in turn send enhanced glutamatergic output to BLA neurons. These reciprocal cortico-amygdala connections mediate sustained fear CR. Thick green arrows and red segments indicate potentiated connections. GABA: GABAergic transmission; Glu: Glutamatergic transmission. Note: for simplicity, other neocortical, hippocampal and hypothalamic regions are not shown.

The basic scheme for fear conditioning, reconsolidation, and extinction (Fig. 7, in particular 7 C) proposed by LeDoux^29^, computationally modeled by Anastasio^30^, and recently reviewed by Tovote *et al.*^31^, assumes that thalamic and cortical glutamatergic projections that convey the signals of the CS (e.g., context) associated with US (e.g., pain) converge on pyramidal neurons and GABAergic interneurons in the BLA. Information is then relayed to the medial part of the amygdaloid central nucleus (CEm), which in turn projects to PAG and pre-motor structures that mediate the CR of fear. BLA pyramidal neurons can also relay signals to the medial ITC (ITCm) that in turn inhibit the lateral part of the amygdaloid central nucleus (CEl) projecting to CEm. However, BLA pyramidal neurons can also excite the lateral ITC (ITCl), which inhibit ITCm that in turn inhibit CEl. This network elicits the disinhibition of CEl and the activation of CEm. The pyramidal neurons of layers II-III and V of the IL/PL subregions of mPFC project not only to the BLA but also directly to ITCl and ITCm^16^,^32^. Accordingly, the activation of lateral amygdala pyramidal neurons drives the formation of fear memories^33^, while BLA lesions disrupt fear CR^17^.

Notably, BLA pyramidal neurons arborize within layers II-III of the mPFC^32^. This unique anatomy enables bidirectional communication between the pyramidal neurons of the BLA and those of mPFC. During fear reconsolidation, the enhanced BLA activity triggers the activity of PL pyramidal neurons that synapse within the BLA, thus modulating the expression of fear responses^34^. Conversely, once extinction is reached, PL activity is inhibited^35^, whereas IL activity is stimulated^36^,^37^. The IL pyramidal neurons involved in the fear response after extinction can suppress CEm responses via BLA GABAergic interneurons and ITC^37^,^38^. However, experimental studies of the involvement of IL in extinction have yielded inconsistent results. Although IL lesions fail to impair extinction recall^39^, optogenetic silencing of IL has no effect^40^, or even facilitates extinction^41^. Interestingly, optogenetic activation of IL has no effect on fear expression that is not yet extinguished, thereby suggesting that IL activation alone is not sufficient to suppress fear expression^41^. The findings of the current study concur with previous findings that CS-evoked IL firing is greater in rodents that fail to acquire extinction than in rodents that do^42^. Moreover, inactivation of the mPFC immediately before extinction facilitates rather than impairs extinction^43^.

In the BLA, GABAergic interneurons (which inhibit glutamatergic pyramidal neurons) are the only ones to contain CB_1_ receptors, which mediate retrograde signaling and depolarization-induced suppression of inhibition. Thus, amygdaloid CB_1_ receptor activation efficiently inhibits GABA release, controlling the efficacy of its own synaptic input in an activity-dependent manner^19^. As we previously demonstrated^11^, AV and AP animals have increased CB_1_ density and functionality in the amygdala. Remarkably, greater CB_1_ availability in the amygdala is associated with increased severity of trauma-related psychopathology, thereby suggesting a key role for compromised endocannabinoid function in endophenotypic and phenotypic expression of threat symptomatology in humans^21^. Notably, mutant mice lacking CB_1_ receptors on GABAergic neurons emit mainly active fear responses (escape attempts and risk assessment), in contrast to mice lacking CB_1_ receptors on glutamatergic neurons, which emit only passive fear responses (freezing) during the extinction process^44^. Despite the current study being unable to directly test the link between CB_1_ receptor expression and behavioral and neurobiological correlates, by considering as a whole the ensemble of our studies regarding this model^10^,^11^,^12^,^13^, it can be hypothesized that in AV and AP mice, the disinhibition of BLA pyramidal neurons is potentiated (Fig. 7D). Because some BLA pyramidal neurons transfer the conditioned fear signals from the intra-amygdaloid circuit to mPFC^45^, it can be reasonably supposed that in AV and AP phenotypes, the increased excitatory activity of BLA pyramidal neurons is associated with increased output to mPFC pyramidal neurons. Indeed, increases in sEPSC frequency and spine density in layer II-III mPFC pyramidal neurons were found only in the AV and AP animals. The mPFC pyramidal neurons in turn may send enhanced glutamatergic output to BLA neurons and thereby increase spinogenesis of BLA neurons, as found in AV and AP mice. We propose that the impaired fear extinction of AV and AP mice is associated with the increased disinhibition of BLA pyramidal neurons.

The lack of extinction, increase of excitatory neurotransmission, and increase of spinogenesis in BLA and mPFC pyramidal neurons of AV and AP mice are highly reminiscent of sensitization by stressful encounters that favors fear responses, sustained neuronal hyperexcitability, increased dendritic branching and spinogenesis in BLA pyramidal neurons^46^,^47^. Stress-induced extinction deficits are associated with dysmorphic mPFC pyramidal neurons, decreased NMDA receptor expression, and altered cue-evoked IL firing^48^,^49^. Thus, extinction-impaired AV and AP mice exhibit modifications in the same BLA-mPFC loop that was made dysfunctional by stress, thereby shifting the balance of the fear matrix to favor the pro-fear pole over the pro-extinction pole^45^. Considered as a whole, our behavioral, electrophysiological and morphological findings demonstrate that the impaired fear extinction of AV and AP animals is related to increased activation of the BLA-mPFC network. Such increased excitatory activity could be linked to a sort of entrapment in the retrieval/reconsolidation process.

Because all same-sex members of inbred strains are genetically identical, the individual differences we observed must reflect allelic and functional differences that are probably controlled by epigenetic factors, such as maternal care or social hierarchy^50^. Based upon the *a priori* individuation of phenotypes (independently of fear conditioning testing), we suggest that the inability to extinguish fear memories is associated with specific neuronal encoding in the fear matrix. Interestingly, healthy monozygotic twins of human patients affected by anxiety-related disorders exhibit fear circuit functional alterations that represent a risk factor for psychopathology^51^. Hyperactivation of the amygdala in relation to attentional bias to threat has been found in individuals with stress- and anxiety-related disorders^27^,^52^. Furthermore, a less-functional endocannabinoid-related gene variation is associated with reduced risk for PTSD and rapid amygdala habituation to threatening stimuli^53^,^54^.

Collectively, our data propose that in avoiding or approaching mice, the improper inhibition of fear is associated with increased spinogenesis and excitatory signaling in the BLA and mPFC. Exposure to risk factors in individuals already at risk owing to their genetic and neurobiological constitution is likely to induce anxiety- or stress-related behaviors. On a brighter note, given that impaired fear extinction may be reversible^55^, investigating its neural mechanisms may open important opportunities for drug discovery and development of next-generation therapies (diet or exposure-based therapy) that could reverse impairments in extinction.

## Methods

### Experimental procedure

The methods were carried out in accordance with the approved guidelines.

Male C57BL/6JOlaHsd mice (n = 169; approximately 40 days old at study onset) (Harlan, Italy) were used. All animals were housed 4 per cage, with food (Mucedola, Italy) and water *ad libitum*. The mice were kept under a 12-h light/dark cycle, controlled temperature (22-23 °C), and constant humidity (60 ± 5%). All efforts were made to minimize animal suffering and reduce the number of animals used, per the European Directive (2010/63/EU). All procedures were approved by the Italian Ministry of Health.

Basing on extreme or mean distribution values of behavioral responses, we selected AV (n = 10), BA (n = 10) and AP (n = 10) animals by testing 169 mice in the Approach/Avoidance (A/A) Conflict Task^10^,^11^,^12^. After two weeks, AV, BA and AP animals were submitted to CFC with repetitive (days 1^st^, 2^nd^, 3^rd^, 7^th^, 14^th^, 21^st^, 28^th^, 60^th^) extinction sessions.

At day 14^th^ of CFC, half of the mice (5 mice/phenotype) were sacrificed to record sEPSCs from mPFC pyramidal neurons by whole cell patch-clamp electrophysiological recordings. At the end of CFC (day 60^th^) the remaining animals were sacrificed for morphological analyses of pyramidal neurons in the BLA and mPFC (PL-IL) using the Golgi-Cox technique. The timing of this double methodological approach was designed to investigate the plastic properties of cortical and amygdaloid pyramidal neurons in relation to middle- and long-term fear extinction.

### Behavioral analyses

#### A/A Conflict Task

The test has been previously described^10^,^11^,^12^. The apparatus consisted of a Plexiglas Y-maze with a starting gray arm from which two arms (8 cm wide, 30 cm long and 15 cm high) stemmed, arranged at an angle of 90° to each other. A T-guillotine door was placed at the end of the starting arm to prevent backward movements of the animal. An arm entry was defined as four legs entering one of the arms. The two choice arms differed in both color and brightness. One of the two arms had a black and opaque floor and walls and no light inside, while the other had a white floor and walls and was lit by a 16-W neon lamp. Notably, the colored “furniture” and the neon lamp were exchangeable between arms to alternate the spatial positions of the white and black arms. The apparatus was placed in a room that was slightly lit by a red light (40 W) and it was always cleaned thoroughly with 70% ethanol and dried after each trial to remove scent cues. At the end of each choice arm, there was a blue chemically inert tube cap (3 cm in diameter, 1 cm deep) that was used as a food tray. The depth of the tray prevented mice from seeing the reward at a distance but allowed easy access to the reward (i.e., eating as well as the appreciation of reward scent, not reducing the olfactory cues).

Because appetites for palatable foods have to be learned, a week before testing the animals were exposed in their home cages for three days to a novel palatable food (Fonzies, KP Snack Foods, Munchen, Germany)^56^. Fonzies (8% protein, 33% fat and 53% carbohydrate, for a caloric value of 541 kcal/100 gm) consisted of corn flour, hydrogenate vegetable fat, cheese powder and salt.

At the beginning of behavioral testing, mice were subjected to 1-day habituation phase in which the Y-Maze arms were opened to encourage maze exploration. During habituation, no food was present in the apparatus. At the end of this phase and during successive testing, to increase the motivation to search for the reward, the animals were slightly food deprived by limiting food access to 12 hours/day. This regimen resulted in no significant body weight loss, as indicated by body weight measures performed at the end of the habituation phase and before testing.

The testing phase started 24 h after the habituation phase at 8 a.m. and consisted of two 10-trial sessions. In Session 1 (S1), the slightly food-deprived animal was placed in the starting stem and could choose to enter one of the two arms, both containing the same standard food reward. After eating, the animal was allowed to stand in its cage for a 1 min-inter-trial interval. At the end of each trial, the reward was replaced. The spatial position of each arm (black and dark or white and lighted) was side balanced during the whole test, to exclude any side preference.

During Session 2 (S2), which started 24 h after S1, the white arm was rewarded with the highly palatable food (Fonzies), while the black arm remained rewarded with the standard food. Notably, the A/A test required to choose between two conflicting drives: reaching a palatable reward placed in an aversive (white and lighted) environment; or reaching a standard food placed in a reassuring (black and dark) environment.

The A/A conflict index represents the difference in the number of white choices between S1 and S2. Given that this index was normally distributed (mean = Δ + 1, SD =  ± 1.7), it allowed us to identify three categories of mice: AV, BA and AP animals. In particular, BA animals reacted with balanced responses between approach and avoidance behaviors and their values in the A/A conflict index corresponded to the mean of the distribution. The two opposite tails of the distribution curve represented the few subjects that exhibited responses that were unbalanced toward one of the conflicting inputs: AV animals had values of the A/A conflict index corresponding to minus two standard deviations of the mean, while AP animals had values corresponding to plus two standard deviations of the mean. This test was implemented only to select animal phenotypes (Fig. 1A).

#### CFC

The test has been previously described^25^ and its details are provided in the Supplementary Information. Briefly, during the Conditioning phase, a mouse was allowed to explore the conditioning chamber (Ugo Basile, Italy) for 3 min. Subsequently, three foot-shocks (0.5 mA, 2.0 s, 1 min inter-shock interval), which represent the US, were emitted. One minute after the third foot-shock, the animal was returned to its home cage.

After 24 h, during the Retrieval phase, and on testing days 2^nd^, 3^rd^, ^7th^, 14^th^, 21^st^, 28^th^ and 60^th^ (i.e., the Extinction phase), the mouse was placed again for 6 min into the conditioning chamber, which represented the CS. No shocks were delivered during the Retrieval and Extinction phases. Notably, during the Retrieval phase, re-exposure to the CS previously paired with the aversive US induced a strong freezing response that progressively declined owing to extinction processes^29^. Freezing times during the first 3 min for each phase were compared among phenotypes.

### Slice preparation and electrophysiological recordings

Electrophysiological protocols have been previously described^57^ and details are provided as Supplementary Information. Briefly, at CFC day 14^th^, the brains of 15 mice (5 mice/phenotype) were cut in 250 μm coronal sections, incubated in oxygenated ACSF, transferred to a recording chamber and submerged in continuously flowing oxygenated ACSF for electrophysiological recordings.

To study the sEPSCs using a Cs-methanesulfonate internal solution, whole-cell patch-clamp recordings in voltage clamp mode (holding potential −70 mV) were performed from mPFC pyramidal neurons, visually identified in slices using an upright infrared microscope (Axioskop 2 FS, Zeiss, Germany), a 40× water-immersion objective (Achroplan, Zeiss, Germany), and a CCD camera (Cool Snap, Photometrics, AZ, USA).

In some experiments the AMPA/Kainate receptor antagonist CNQX (10 μM) and the NMDA receptor antagonist AP-5 (50 μM) were added to the ACSF. In this condition all synaptic events were blocked, indicating that they were due to glutamatergic receptor activation^57^.

Spontaneous synaptic events were analyzed off-line using the Mini Analysis Program (Synaptosoft Inc., USA). sEPSCs were manually detected using a 10 pA threshold crossing algorithm. The inter-event interval, event amplitude and kinetic parameters (rise and decay times) were compared among phenotypes.

### Labeling of Recorded Cells

The cell labeling protocol has been previously described^57^ and details are provided in the Supplementary Information. Briefly, to morphologically identify the recorded cells, some neurons were filled with 2% biocytin (Sigma-Aldrich, Saint Louis, MO, USA) through the recording pipette. Immediately after recording, slices containing biocytin-loaded cells were incubated with Cy2-conjugated Streptavidin (1:200; Jackson Immunoresearch Laboratories, PA, USA). To assess the cytoarchitectonic areas and layers, slices were counterstained with NeuroTrace® 640⁄660 deep-red Fluorescent Nissl Stain (Invitrogen) and mounted using an anti-fade medium (Fluoromount; Sigma-Aldrich, Saint Louis, MO, USA). Neurons of interest were identified using a 10× objective (Plan-Apochromat, Zeiss, Germany) and captured on a 40X oil immersion objective through a confocal laser-scanning microscope (CLSM700; Zeiss, Germany).

### Morphological analyses

#### Golgi-Cox technique

Morphological analyses have been previously described^58^, and details are provided in the Supplementary Information. Briefly, at the end of the CFC (day 60^th^), the brains of 15 mice (5 mice/phenotype) were processed by the Golgi-Cox technique to analyze neuronal morphology (dendritic length and nodes, spine density) in 200 μm coronal slides at the level of the BLA (from − 0.70 to −2.06 mm in relation to bregma) and mPFC (PL cortex: from + 2.96 to 1.42; IL cortex: from + 2.10 to 1.34 mm)^59^. The coronal sections were stained according to the method described by Gibb and Kolb^60^. The stained sections were analyzed by using a light microscope (Axioskop 2, Zeiss, Germany) with a 100X oil-immersion objective lens. A researcher unaware of the specimen phenotype performed morphological analyses using Neurolucida v 11 (MicroBrightField, VT, USA) software to reconstruct dendritic arbors.

### Morphological features of mPFC - BLA pyramidal neurons

mPFC pyramidal neurons were identified by the presence of: a typical conic soma; a distinct single apical dendrite arising from the vertex of the soma, coursing toward the pial surface and entering molecular layer I; several basilar dendrites arising from the base of the soma, thinner and shorter than those of the apical arborization; and dendritic spines along both apical and basal arborizations. According to these criteria, 30 pyramidal neurons (2 neurons per animal for a total of 10 neurons per phenotype) belonging to layer II-III of IL/PL mPFC were selected.

BLA “pyramidal” neurons encompass a broad, continuous morphological spectrum, from neurons that are virtually identical to cortical pyramidal neurons to neurons that closely resemble cortical spiny stellate cells. Furthermore, bitufted/bipolar cells are present. According to these criteria, 30 pyramidal neurons (2 neurons per animal for a total of 10 neurons per phenotype) belonging to the BLA were selected.

In each selected neuron, the apical and basal dendritic trees were separately examined using Sholl Analysis. The parameters analyzed were: dendritic length (in μm), calculated by summing the length of all processes passing through each shell; dendritic nodes, calculated by summing all points from which dendritic branches arose; terminal spine density (spine density/25 μm), calculated by measuring a 25 μm length of the dendritic terminal and counting the number of spines along the segment.

### Statistical analyses

All data were tested for normality (Will-Shapiro’s test) and homoscedasticity (Levene’s test). Statistical analyses were applied on a per mouse basis. Behavioral and morphological data were compared using ANOVAs, followed by Newman-Keuls tests when appropriate. Electrophysiological data were compared by using ANOVAs and Kolmogorov-Smirnov (K-S) tests. Values of P < 0.05 were considered statistically significant.

## Additional Information

**How to cite this article**: Laricchiuta, D. *et al.* Maintenance of aversive memories shown by fear extinction-impaired phenotypes is associated with increased activity in the amygdaloid-prefrontal circuit. *Sci. Rep.* **6**, 21205; doi: 10.1038/srep21205 (2016).

## Supplementary Material

Supplementary Information
