# Supplementary material for: Maintenance of aversive memories shown by fear extinction-impaired phenotypes is associated with increased activity in the amygdaloid-prefrontal circuit
Source: Sci Rep. 2016 Feb 15;6:21205. doi: 10.1038/srep21205 (PMC4753413; doi:10.1038/srep21205)
Supplement: Supplementary Information [file srep21205-s1.doc]

Maintenance of aversive memories shown by fear extinction-impaired phenotypes is associated with increased activity in the amygdaloid-prefrontal circuit

Daniela Laricchiuta^a,b,*^, Luana Saba^a,c^, Paola De Bartolo^a,d^, Silvia Caioli^a,c^, Cristina Zona^a,c^,

Laura Petrosini^a,b^

# ^a^ IRCCS Fondazione Santa Lucia, Rome, Italy

# ^b^ Department of Psychology, University Sapienza of Rome, Rome, Italy

# ^c^ Department of Neuroscience, University of Rome "Tor Vergata", Rome, Italy

^d^ Department of Sociological and Psychopedagogical Studies, University Guglielmo Marconi of Rome, Rome, Italy

**^*^ Corresponding author:**

Daniela Laricchiuta,

^1^ IRCCS Fondazione Santa Lucia,

via del Fosso di Fiorano 64, 00143, Rome, Italy

E-mail address: daniela.laricchiuta@uniroma1.it

**CFC**

The task was carried out in a soundproof conditioning chamber (50 cm long, 24.5 cm wide, 26.5 cm high) (UgoBasile, Italy) made of gray Perspex with a metal grid floor. A video camera placed above the conditioning chamber allowed observing animal behavior. At the first day (Conditioning phase), each mouse was allowed exploring the conditioning chamber for 3 min. Three foot-shocks (0.5 mA, 2.0 s, 1 min inter-shock interval) representing the unconditioned stimulus (US) were then emitted. After 1 min from the third foot-shock, the animal was removed from the chamber and returned to its home cage. The employed foot-shock parameters evoke signs of discomfort as flinching, jumping, and vocalizing, as well as the freezing behavior, reliable index of aversive memories.

Twenty-four hours after conditioning (Retrieval phase), and at days 2^nd^, 3^rd^, 7^th^, 14^th^, 21^st^, 28^th^ and 60^th^ (Extinction phase), the mouse was placed back in the chamber representing the conditioned stimulus (CS) for 6 min. No shocks were delivered during Retrieval and Extinction phases. Notably, in rodents during the Retrieval phase re-exposure to the CS previously paired with the aversive US induces a strong freezing response, represented by the lack of movements except those associated with breathing. As CS exposure continues, the freezing rate progressively declines due to extinction and/or habituation processes. Freezing was recorded by an experimenter blind to the phenotype the animal belonged to and freezing times during the first 3 min for Conditioning, Retrieval and Extinction phases were compared among phenotypes.

**Slice preparation and electrophysiological recordings**

At day 14^th^ of CFC, AV (n=5), BA (n=5), and AP (n=5) mice were anaesthetized with halothane (Sigma-Aldrich, Saint Louis, [MO](https://www.google.it/search?espv=2&biw=1920&bih=940&q=missouri&stick=H4sIAAAAAAAAAGOovnz8BQMDgzMHnxCnfq6-gYWlRXKlEgeIaVKZnKGllZ1spZ9flJ6Yl1mVWJKZn4fCscpITUwpLE0sKkktKjZnDLnFebzk_ru181eJP_jqzxZ4rQsAy63OEGAAAAA&sa=X&ei=oKyaVYblF8iesgHq463YBA&ved=0CIwBEJsTKAIwEA), USA) and decapitated. Brains were rapidly removed and cut in 250 μm thick coronal sections with a vibratome (Leica VT1000S, Wetzlar, Germany) in ice-cold artiﬁcial cerebrospinal ﬂuid solution (ACSF) containing (in mM): 126 NaCl, 26 NaHCO_3_, 2.5 KCl, 1.25 NaH_2_PO_4_, 2 MgSO_4_, 2 CaCl_2_, and 10 glucose, gassed with 95% O_2_–5% CO_2_ (pH 7.4, 300 mOsm). Slices were incubated in the oxygenated ACSF for 30 minutes at 37°C, subsequently at room temperature (RT), and then transferred to a recording chamber and submerged in continuously flowing oxygenated ACSF (31°C, 2 mL/min) for electrophysiological recordings.

Whole-cell patch-clamp recordings in voltage clamp mode (holding potential -70 mV) were performed from medial prefrontal cortex (mPFC) pyramidal neurons, visually identified in slices using an upright infrared microscope (Axioskop 2 FS, Zeiss, Germany), a 40X water-immersion objective (Achroplan, Zeiss, Germany), and a CCD camera (Cool Snap, Photometrics, AZ, USA).

To study spontaneous excitatory postsynaptic currents (sEPSCs), whole-cell recording electrodes were pulled from borosilicate glass pipettes (outside diameter 1.5 mm; tip resistance 3-4 MΩ) and then filled with an intracellular solution containing (in mM): 139.5 cesium methanesulfonate (CsMeSO_3_), 1 ethylene glycolbis (β-aminoethyl ether)-*N,N,N*’*,N*’-tetraacetic acid (EGTA), 5.5 CsCl_2_, 0.1 CaCl_2_, 10 *N*-2-hydroxyethylpiperazine-*N*’-2-ethanesulfonic acid (HEPES), 2 MgCl_2_, 2 Mg-ATP, (pH 7.3, 290 mOsm). By using ACSF and this internal solution, the chloride equilibrium potential, calculated by the Nernst equation, was -70 mV. Under these experimental conditions, the net flux of chloride ions was null, ensuring that only glutamatergic excitatory currents were recorded. In some experiments the AMPA/Kainate receptor antagonist 6-cyano-7-nitroquinoxyline-2,3-dione (CNQX, 10 μM), and the NMDA receptor antagonist amino-5-phosphonovaleric acid (AP-5, 50 μM) were added to ACSF.

In order to morphologically identify the recorded cells, some neurons were filled with 2% biocytin (Sigma-Aldrich, Saint Louis, [MO](https://www.google.it/search?espv=2&biw=1920&bih=940&q=missouri&stick=H4sIAAAAAAAAAGOovnz8BQMDgzMHnxCnfq6-gYWlRXKlEgeIaVKZnKGllZ1spZ9flJ6Yl1mVWJKZn4fCscpITUwpLE0sKkktKjZnDLnFebzk_ru181eJP_jqzxZ4rQsAy63OEGAAAAA&sa=X&ei=oKyaVYblF8iesgHq463YBA&ved=0CIwBEJsTKAIwEA), USA) through the recording pipette.

After the formation of a high-resistance seal (>1GΩ), capacitance and resistance of electrodes were compensated electronically. Signals were ampliﬁed by using a Multiclamp 700B patch-clamp ampliﬁer, sampled at 20 KHz, ﬁltered at 3 KHz, and stored in a computer. Data were acquired with a Digidata 1400A (Molecular Devices, CA, USA). Capacitance and resistance membrane values were obtained by using the Membrane Test Function of pClamp 10 software (Molecular Devices, CA, USA). Whole-cell access resistances measured in voltage-clamp mode were in the range of 5–20 MΩ. Spontaneous synaptic events were analysed off-line by using the Mini Analysis Program (Synaptosoft Inc., Decatur, GA, USA). sEPSCs were manually detected by using a 10 pA threshold crossing algorithm. Inter-event interval, event amplitude and kinetic parameters (rise and decay times) were compared among phenotypes.

**Labelling of Recorded Cells**

Immediately after recording, the slices containing biocytin loaded cells were fixed by immersion in 4% paraformaldehyde in 0.1 M PBS overnight at 4°C. The slices were collected in PBS, rinsed 3 times in the same buffer, and then incubated with Cy2-conjugated Streptavidin (1 : 200; Jackson Immunoresearch Laboratories, PA, USA) in PBS 0.3% Triton X-100 for 3 h at RT.

Furthermore, to assess the cytoarchitectonic areas and layers of the different recorded slices, the latter were counterstained with NeuroTrace® 640⁄660 deep-red Fluorescent Nissl Stain (Invitrogen). After additional 3 washes in PBS, the sections were mounted using an anti-fade medium (Fluoromount; Sigma-Aldrich, Saint Louis, [MO](https://www.google.it/search?espv=2&biw=1920&bih=940&q=missouri&stick=H4sIAAAAAAAAAGOovnz8BQMDgzMHnxCnfq6-gYWlRXKlEgeIaVKZnKGllZ1spZ9flJ6Yl1mVWJKZn4fCscpITUwpLE0sKkktKjZnDLnFebzk_ru181eJP_jqzxZ4rQsAy63OEGAAAAA&sa=X&ei=oKyaVYblF8iesgHq463YBA&ved=0CIwBEJsTKAIwEA), USA). Neurons of interest were identified on 10X objective (Plan-Apochromat, Zeiss, Germany; NA = 0.30) and captured on 40X oil immersionobjective (NA = 1.3; zoom factor 0.5) through a confocal laser scanning microscope (CLSM700; Zeiss, Germany). The following acquisition settings were used: image format 1024 × 1024; image size 318.8 × 319.8 μm; Airy units 1.55 producing an optical section thickness of 1.2 μm; 25 z-stack series with az-spacing 1.25 μm; pixel dwell time 3.15 μs. To generate projection images, the maximum intensity algorithm of the CLSM software was used. The confocal image acquisitions were performed so that all samples were captured using consistent settings for laser power and detector gain. Finally, the figures were generated by adjusting only the brightness and the contrast and were composed using Adobe Illustrator CS5.

**Morphological analyses**

**Golgi-Cox technique**

At the end of CFC (day 60^th^), the remaining mice were sacrificed under deep anesthesia, transcardially perfused with saline, brains were removed and tissues processed with Golgi-Cox technique for analyzing neuronal morphology (neuronal size, dendritic length and spine density) in 200 μm coronal slides of basolateral amygdala (BLA) and mPFC. Briefly, brains were immersed in a solution performed with 5% Cr_2_K_2_O_7_, 5%Cl_2_ Hg, and 5% CrK_2_O_4_ (Sigma-Aldrich, Saint Louis, [MO](https://www.google.it/search?espv=2&biw=1920&bih=940&q=missouri&stick=H4sIAAAAAAAAAGOovnz8BQMDgzMHnxCnfq6-gYWlRXKlEgeIaVKZnKGllZ1spZ9flJ6Yl1mVWJKZn4fCscpITUwpLE0sKkktKjZnDLnFebzk_ru181eJP_jqzxZ4rQsAy63OEGAAAAA&sa=X&ei=oKyaVYblF8iesgHq463YBA&ved=0CIwBEJsTKAIwEA), USA) in distilled water for 10 days, transferred to a 30% sucrose solution for 3-5 days, and then sectioned at the level of mPFC (considering prelimbic cortex from + 2.96 to 1.42, and infralimbic cortex from + 2.10 to 1.34 mm in relation to bregma) and BLA (from - 0.70 to - 2.06 mm in relation to bregma) by using a vibratome. The coronal sections were mounted on gelatinized slides and stained. Slides were rinsed in distilled water for 1 min, placed in H_5_NO (Sigma-Aldrich, Saint Louis, [MO](https://www.google.it/search?espv=2&biw=1920&bih=940&q=missouri&stick=H4sIAAAAAAAAAGOovnz8BQMDgzMHnxCnfq6-gYWlRXKlEgeIaVKZnKGllZ1spZ9flJ6Yl1mVWJKZn4fCscpITUwpLE0sKkktKjZnDLnFebzk_ru181eJP_jqzxZ4rQsAy63OEGAAAAA&sa=X&ei=oKyaVYblF8iesgHq463YBA&ved=0CIwBEJsTKAIwEA), USA) for 30 min in the dark, rinsed in distilled water for 1 min, placed in Kodak Fix (Sigma-Aldrich, Saint Louis, [MO](https://www.google.it/search?espv=2&biw=1920&bih=940&q=missouri&stick=H4sIAAAAAAAAAGOovnz8BQMDgzMHnxCnfq6-gYWlRXKlEgeIaVKZnKGllZ1spZ9flJ6Yl1mVWJKZn4fCscpITUwpLE0sKkktKjZnDLnFebzk_ru181eJP_jqzxZ4rQsAy63OEGAAAAA&sa=X&ei=oKyaVYblF8iesgHq463YBA&ved=0CIwBEJsTKAIwEA), USA) for film for 30 min in the dark, rinsed in distilled water for 1 min, placed sequentially in 50, 70, and 95% alcohol for 1 min, twice in 100% alcohol for 5 min, in a solution of one-third chloroform, one-third xylene, and one-third 100% alcohol for 15 min, and then placed in xylene for 15 min. Finally, the sections were cover slipped with Canada Balsam.

The stained sections were analyzed by using a light microscope (Axioskop2, Zeiss, Germany) with a 100X oil-immersion objective lens. A researcher unaware of the phenotype of the specimen performed morphological analyses by using the Neurolucida v 11 (MicroBrightField, VT, USA) software for reconstructing dendritic arbors. Neurons were selected only if the labeling was uniform and lacked any reaction precipitate, they were relatively isolated from neighboring impregnated neurons to avoid overlapping, the predominant plane of the dendritic arbors was parallel to the plane of the section, the dendritic arborizations were intact and visible as far as the most distal branches of apical and basal dendrites, and spines were clearly marked. In each selected neuron, apical and basal dendritic trees were separately examined by Sholl Analysis. This procedure is based on virtually including the whole neuron in a set of concentric shells at 10 µm intervals centered on the cell body. The parameters analyzed were: dendritic length (in μm), calculated by summing the length of all processes passing through each shell; dendritic nodes, calculated by summing all points from which dendritic branches arose; terminal spine density (spine density/25µm), calculated by measuring the length of dendritic terminal of 25 µm and counting the number of spines (i.e., the protrusions of the dendritic membrane regardless of their shape or actual function) along the segment.
